# Risk factors for invasive fungal infections after kidney transplantation and the construction of a predictive model

**DOI:** 10.3389/fmed.2026.1850921

**Published:** 2026-10-12

**Authors:** Lulu Tang, Jiqiu Wen, Hongdi Xiong, Chunying Lv, Jianhui Dong, Ning Wen, Guomei Huang, Zhiwen Jiang, Haiju Chen, Cairong Yan, Shuangshuang Li, Yuyang Jiang, Yi Wu, Cunwei Cao, Xuyong Sun

**Affiliations:** 1 Department of Dermatology and Venereology, the First Affiliated Hospital of Guangxi Medical University, Nanning, China; 2 Department of Dermatology and Venereology, the Second Affiliated Hospital of Guangxi Medical University, Nanning, China; 3 Institute of Transplant Medicine, The Second Affiliated Hospital of Guangxi Medical University, Nanning, China; 4 Guangxi Clinical Research Center for Organ Transplantation, Nanning, China; 5 Guangxi Key Laboratory of Organ Donation and Transplantation, Nanning, China; 6 Guangxi Scientific and Technological Innovation Cooperation Base of Mycosis Prevention and Control, Nanning, China; 7 Guangxi Key Laboratory of Mycosis Prevention and Treatment, Nanning, China

**Keywords:** high-risk factors, infection, invasive fungal infection, kidney transplantation, prediction model

## Abstract

**Background:**

Post-transplant infection is one of the main complications in the early stage of kidney transplantation. However, there is a lack of risk prediction tools to identify those at high risk of infection.

**Objective:**

To investigate the risk factors for invasive fungal infections (IFIs) after kidney transplantation in a single center in Guangxi region and to construct a predictive model.

**Methods:**

A total of 997 patients who underwent kidney transplantation were selected as the research subjects. Patients with IFIs after kidney transplantation were classified into the IFIs group (*n* = 125), while the remaining patients formed the control group (*n* = 872). The clinical data of the two groups were compared, and the risk factors for IFIs after kidney transplantation were analyzed using multivariate logistic stepwise forward regression. The 997 samples were randomly divided into a training set and a validation set in an 7:3 ratio. The training set was used to build the model, while the validation set was used to evaluate the model performance.

**Results:**

Heart failure (OR = 4.235), low EOS% level (OR = 0.825), high Cr level (OR = 1.003), and low Mg + level (OR = 0.198) are independent risk factors influencing the occurrence of IFIs after kidney transplantation (all *P* < 0.05). Based on these four risk factors, a nomogram prediction model was constructed. The AUC of the training set was 0.654 (95% CI: 0.591–0.717), with a specificity of 59.3% and a sensitivity of 65.3%. The AUC of the validation set was 0.677(95% CI: 0.583–0.770), with a specificity of 59.0% and a sensitivity of 76.6%.

**Conclusion:**

Heart failure, low EOS%, high Cr level, and low Mg level are closely related to the occurrence of IFIs after kidney transplantation. The prediction model based on these risk factors has certain predictive value.

## 1 Introduction

End-stage renal disease (ESRD) is a major chronic disease globally, posing a significant threat to human health and well-being (1). Kidney transplantation is considered the preferred treatment for ESRD patients, as it can significantly improve the quality of life and prognosis of patients. However, post-transplant infection is one of the main complications in the early stage of kidney transplantation, and it is also an important factor contributing to the early death of kidney transplant recipients (2). After kidney transplantation, the use of high-concentration immunosuppressants can, to a certain extent, suppress the immune response of the body, but it also significantly weakens the body's defense ability, thereby increasing the risk of post-transplant infection (3). Studies have shown that within one year after kidney transplantation, 45.0% to 60.2% of patients will experience infection; moreover, the risk of death due to infection after kidney transplantation is 32 times that of healthy individuals (4, 5). Severe infection not only damages the function of the transplanted kidney but also significantly increases the postoperative mortality rate of patients, posing a serious challenge to solid organ transplantation (6).

The types of infections after kidney transplantation are diverse, including bacterial, viral, and fungal infections. Among them, invasive fungal infections (IFIs) are one of the most serious types of infections after kidney transplantation. Although the incidence of IFIs is relatively low, its mortality rate is extremely high (7). Moreover, the clinical manifestations of IFIs are often atypical, making early diagnosis difficult, which further increases the complexity and challenge of treatment (8). Given the complex and diverse high-risk factors for infectious complications after kidney transplantation (9, 10), the construction of a predictive model is of great significance for the early identification of high-risk patients and the optimization of prevention and treatment strategies. Currently, there have been studies in China attempting to build predictive models for infections after kidney transplantation, but there are relatively few specialized studies in the Guangxi region. Specialized predictive models for IFIs still need to be further developed and improved. Therefore, this article intends to explore the risk factors for IFIs after kidney transplantation in a single center in the Guangxi region and construct relevant predictive models, in order to provide scientific basis and precise preventive measures for reducing postoperative infections.

## 2 Materials and methods

### 2.1 Study subjects

A retrospective collection of patients who underwent kidney transplantation at the Second Affiliated Hospital of Guangxi Medical University from January 2021 to September 2023 was used as the research subjects. According to the inclusion and exclusion criteria, a total of 997 recipients who underwent kidney transplantation were finally included. This study was approved by the Medical Ethical Committee of the Second Affiliated Hospital of Guangxi Medical University (2025-KYL(077), and all methods were carried out in accordance with the 1964 Helsinki Declaration and its later amendments or comparable ethical standards. Since this study is retrospective and the data used did not involve any personally identifiable information, the need to obtain the informed consent was waived by the Second Affiliated Hospital of Guangxi Medical University Medical Ethical Committee.

Inclusion criteria: (1) Patients who received their first kidney transplantation surgery; (2) Patients with follow-up time; (3) Donated organs from deceased donors.

Exclusion criteria: (1) Patients with missing clinical data or lost to follow-up; (2) Patients undergoing multi-organ combined transplantation.

### 2.2 Data collection

Clinical data of patients were collected through the hospital's electronic medical record system, including receptor factors, laboratory test results, treatment factors, and postoperative infection conditions, etc.

Receptor factors: General information such as gender, age, blood type, body mass index (BMI), previous medical history (including Coronary artery disease, Hypertension, Diabetes, Anemia, Hyperlipemia, Heart failure, Long-term use of calcineurin inhibitors, Combined with malignant tumor), and preoperative dialysis duration.

Laboratory test results: For patients who developed IFIs during hospitalization, laboratory indicators were collected prior to infection onset; for those who developed IFIs after discharge, laboratory data were obtained at the last assessment before discharge. (1) Blood routine indicators include white blood cell count (WBC), red blood cell count (RBC), hemoglobin (HGB), platelet count (PLT), neutrophil percentage (NEU%), lymphocyte percentage (LYM%), monocyte percentage (MONO%), eosinophil percentage (EOS%), basophil percentage (BASO%), hematocrit (HCT), mean red blood cell volume (MCV), mean red blood cell hemoglobin content (MCH), mean red blood cell hemoglobin concentration (MCHC), etc.; (2) Liver and kidney function indicators include total bilirubin (TBIL), direct bilirubin (DBIL), indirect bilirubin (IBIL), total protein (TP), albumin (ALB), globulin (GLOB), albumin-to-globulin ratio (A/G), glutamyl transpeptidase (GGT), aspartate aminotransferase (AST), alanine aminotransferase (ALT), alkaline phosphatase (ALP), urea nitrogen (BUN), serum creatinine (Cr), uric acid (UA), etc.; (3) Electrolyte indicators include potassium (K), sodium (Na), chloride (Cl), calcium (Ca), magnesium (Mg), etc.; (4) T lymphocyte subset indicators include CD3 + T (%), CD4 + T (%), CD8 + T (%), CD4 + T/CD8 + T; (5) Glycogen-lipid metabolism indicators include fasting blood glucose (FPG), total cholesterol (TC), triglycerides (TG), high-density lipoprotein cholesterol (HDL-C), low-density lipoprotein cholesterol (LDL-C).

Treatment factors: Duration of surgery, Regimen of immunosuppressive drugs. All patients underwent immunosuppressive induction. Induction protocols, whether there have been rejection reactions, and whether there have been events such as intensified immunosuppressant therapy.

Postoperative infection conditions: Whether there was infection after transplantation, and the pathogenic bacteria (fungi/bacteria/viruses) causing the infection. The diagnostic criteria are based on the definition of IFD jointly released by the European Organization for Research and Treatment of Cancer/Invasive Fungal Infections Cooperative Group and the National Institute of Allergy and Infectious Diseases Mycoses Study Group, EORTC/MSG.

### 2.3 Surgical procedure and immunosuppressive regimen

The blood types of both donors and recipients were in compliance with the principles of blood transfusion, and there were no contraindications for kidney transplantation. The selection criteria for recipients were in accordance with the technical specifications for kidney transplantation. All deceased organ donors met the norms for organ donation after the death of Chinese citizens.

The immunosuppressive treatment regimens include: ① calcineurin inhibitors (CNI) + mycophenolic acid (MPA) + glucocorticoids; ② CNI + Mizoribine (MZR) + glucocorticoids; ③ CNI + mTOR inhibitors + glucocorticoids. CNI is tacrolimus or cyclosporine; MPA is mycophenolic acid or mycophenolate sodium; glucocorticoids are prednisone or methylprednisolone or methylprednisolone; mTOR inhibitors are sirolimus or everolimus. During the treatment process, the drug concentrations were monitored in a timely manner, and the drug doses were adjusted according to the actual condition of the patients to maintain the blood drug concentration within the therapeutic range. Preventive and therapeutic strategies for early acute organ rejection after kidney transplantation include no treatment, or the use of immunosuppressive agents such as basiliximab, anti-human T-cell porcine immunoglobulin, or rabbit anti-human thymocyte immunoglobulin.

### 2.4 Follow-up

According to the follow-up standards for kidney transplant recipients, outpatient and telephone follow-ups were conducted to regularly test blood and urine routine, liver and kidney functions, lymphocyte immune analysis, immunosuppressant concentrations, and imaging examinations, etc. The follow-up continued until December 31, 2024, and the death status and survival time were recorded.

### 2.5 IFIs diagnosis and grouping

The diagnosis of invasive fungal disease was established based on a combination of host factors, clinical criteria, and mycological evidence.

Host factors included at least one of the following conditions: (1) recent neutropenia (absolute neutrophil count <500 × 10^6^/L) lasting for more than 10 days; (2) receipt of allogeneic hematopoietic stem cell transplantation; (3) prolonged use of corticosteroids for more than 3 weeks at a dose ≥0.3 mg·kg⁻^1^·day⁻^1^ (excluding allergic bronchopulmonary aspergillosis); (4) use of T-cell immunosuppressive agents (e.g., cyclosporine A, tumor necrosis factor-α inhibitors, monoclonal antibodies such as alemtuzumab) or nucleoside analogs within 90 days; (5) history of invasive fungal infection; (6) presence of acquired or inherited immunodeficiency (e.g., AIDS, chronic granulomatous disease, or combined immunodeficiency).

Clinical criteria included at least one of the following: (1) Lower respiratory tract infection: chest CT showing at least one of the following features: (i) dense, well-circumscribed lesions with or without halo sign; (ii) air crescent sign; (iii) cavitary lesions; (2) Tracheobronchitis: bronchoscopic findings of tracheal or bronchial ulceration, nodules, pseudomembranes, plaques, or eschars; (3) Sinonasal infection: at least one of the following: (i) acute localized pain (including radiation to the orbit); (ii) nasal ulceration with black eschar; (iii) bone destruction of the sinus with possible intracranial extension; (4) Central nervous system involvement: at least one of the following: (i) focal lesions on imaging; (ii) meningeal enhancement on MRI or CT; (5) Disseminated candidiasis: candidemia within the previous 2 weeks, accompanied by at least one of the following: (i) “bull's-eye” lesions in the liver or spleen; (ii) progressive retinal exudates on ophthalmologic examination.

Mycological evidence included direct or indirect evidence of fungal infection:

(1) Direct evidence: (i) detection of fungal elements consistent with molds in sputum, bronchoalveolar lavage fluid (BALF), bronchial brushings, or sinus aspirates microscopy, cytology, or culture; (ii) positive culture or microscopic identification of Cryptococcus species in sputum or BALF. (2) Indirect evidence: (i) positive galactomannan antigen test for Aspergillus in plasma, serum, BALF, or cerebrospinal fluid; (ii) positive serum (1,3)-β-D-glucan assay (excluding cryptococcosis and mucormycosis); (iii) positive cryptococcal capsular polysaccharide antigen.

Based on the above criteria, IFIs was classified into the following categories:

1) Confirmed IFIs: Confirmed by histopathological or cytopathological evidence of fungal elements in tissue obtained by needle aspiration or biopsy from a normally sterile site, or by positive culture from a sterile site consistent with clinical or radiological evidence of infection. For *Pneumocystis jirovecii*, identification of cysts or trophic forms in lung tissue, BALF, or sputum was considered diagnostic.
2) Clinically diagnosed IFIs: Defined by the presence of at least one host factor, one clinical criterion, and one item of mycological evidence.
3) Possible IFIs: Defined by the presence of at least one host factor and one clinical criterion, without mycological evidence.
4) Unclassified IFIs: Cases with host factors but insufficient clinical or microbiological evidence to meet the above definitions.

All diagnostic procedures were performed according to standard clinical practice during the study period. IFIs include both confirmed patients (4 cases) and clinically diagnosed patients (121 cases). Based on whether renal transplant recipients developed invasive fungal infections after surgery, patients who did develop the infection were classified into the IFIs group (*n* = 125), while the remaining patients were classified as the control group (*n* = 872). The control group consisted of all kidney transplant recipients who did not develop IFIs during the follow-up period, including recipients with bacterial or viral infections.

### 2.6 Construction and evaluation of a predictive model for invasive fungal infections after kidney transplantation

The clinical data of the IFIs group and the control group were compared through univariate analysis, and the indicators with statistically significant differences in the univariate analysis were subjected to multivariate logistic regression analysis.

Random numbers were generated using the “sample” function in R software, and the enrolled patients were randomly divided into a training set and a validation set in an 7:3 ratio. Set the random number seed to 1234. The training set included 697 patients (86 IFI and 611 controls), and the validation set included 300 patients (39 IFI and 261 controls). The training set was used for risk factor analysis and model construction, while the validation set was used to evaluate the predictive efficacy of the model.

Based on the data from the training set, multivariate logistic regression analysis was used to screen out statistically significant indicators as predictive factors, and a nomogram predictive model for invasive fungal infections in kidney transplant recipients after kidney transplantation was constructed using R software and its rms package. To evaluate the stability of the model, internal validation was conducted using the Bootstrap resampling method, with 500 resampling iterations set. The predictive efficacy of the validation set was evaluated using ROC curves, calibration curves, and clinical decision curves.

### 2.7 External validation cohort

An independent external validation cohort was retrospectively collected from the Second Affiliated Hospital of Guangxi Medical University, including 223 kidney transplant recipients admitted between January 2024 and July 2024. The eligibility criteria were identical to those used in the development cohort. Patients were followed until July 2026, and 27 cases of IFIs were identified. The established nomogram was applied directly to the external validation cohort without model modification.

### 2.8 Statistical methods

Statistical analysis was conducted using SPSS 26.0 and R 4.2.1 software. Measurement data were expressed as mean ± standard deviation. For measurement data that followed a normal distribution, *t*-test was used for comparison; for those that did not follow a normal distribution, rank sum test was used. Count data were represented as *n*(%) and compared between groups using *χ*2 test. Missing data were handled using mean imputation because the proportion of missing values was low (<5%) and the missing variables were continuous clinical parameters. Sensitivity analyses were performed to assess the robustness of the results. Variables with significance in univariate analysis were included in the multivariate logistic stepwise forward regression analysis. A difference was considered statistically significant when the two-tailed *P* value was less than 0.05.

## 3 Results

### 3.1 Incidence of IFIs after kidney transplantation

This study included 125 patients with IFIs after kidney transplantation (IFIs group), among whom 83 were male (66.40%) and 42 were female (33.60%), as shown in Figure 1A; 104 cases were single infections (83.20%), and 21 cases were mixed infections (16.80%), as shown in Figure 1C; 110 cases had lung infections (85.93%). A total of 144 fungal strains were detected, including 76 strains of *Pneumocystis jirovecii*, 28 strains of *Candida*, 33 strains of *Aspergillus*, 3 strains of *Trichosporon asahii*, 2 strains of *Talaromyces marneffei*, 1 strain of *Cryptococcus*, and 1 strain of *Purpureocillium lilacinum*, as shown in Figure 1B. There were 103 cases (82.40%) of infection within 1 year after transplantation, 16 cases (12.80%) within 1–2 years after transplantation, and 6 cases (4.80%) within 2–3 years after transplantation, as shown in Figure 1D.

**Figure 1:**
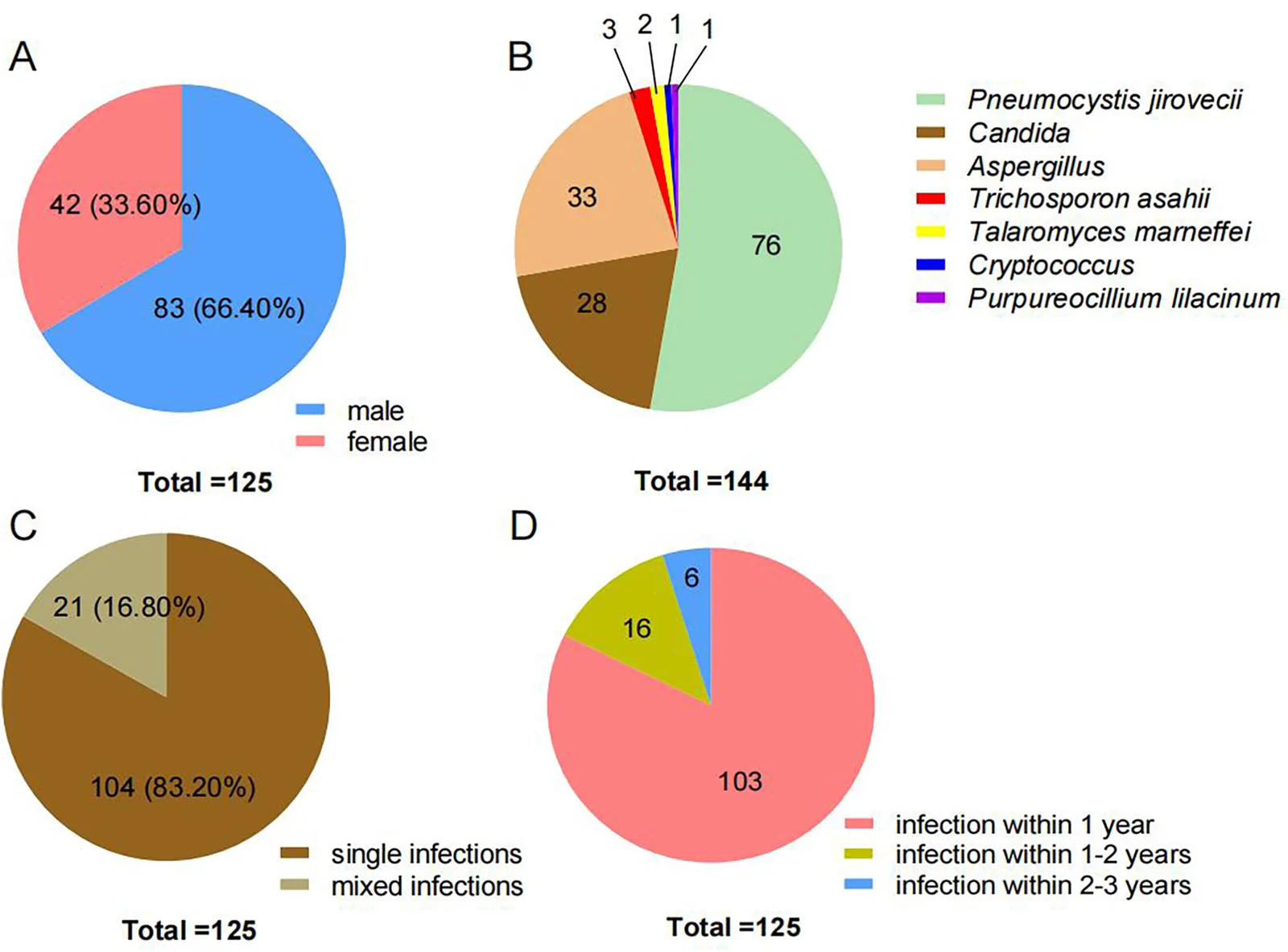
The epidemiological ncharacteristics of IFIs. **(A)** Gender distribution **(B)** Type of infection **(C)** Distribution of infected fungi **(D)** Infection time.

In this study, patients were infected with a variety of fungal pathogens, including *Pneumocystis jirovecii*, *Candida spp.*, *Aspergillus spp.*, *Trichosporon asahii*, *Talaromyces marneffei*, *Cryptococcus spp.*, and *Purpureocillium lilacinum*. Regarding prophylactic treatment, most patients in this cohort received routine post-transplant prophylaxis according to standard clinical practice. Trimethoprim–sulfamethoxazole (TMP-SMX) was commonly prescribed for the prevention of opportunistic infections, typically for 3–6 months. Antifungal prophylaxis was also administered based on individual risk assessment. However, due to the retrospective nature of the study and incomplete medical records, detailed information on medication dosage, duration, and adherence was not consistently available for all patients. In addition, some patients were followed up remotely, and variability in adherence, including self-discontinuation of medications, was observed. Therefore, prophylactic treatment was not included as a variable in the subsequent statistical analysis.

### 3.2 Univariate analysis of factors influencing the occurrence of IFIs after kidney transplantation

By comparing the general data of the two groups of patients, the results showed that the proportion of patients with a history of heart failure before the operation in the IFIs group was significantly higher than that in the control group, and the difference was statistically significant (*P* < 0.05). There were no statistically significant differences in age, gender, blood type, BMI, preoperative dialysis time, operation duration, and immunosuppressive agent regimen between the two groups (*P* > 0.05, Table 1).

**Table 1:** Comparison of general information of the two groups.

Characteristics	Sub-category	IFIs group (*n* = 125)	Control group (*n* = 872)	*χ*^2^/t	*P*
Age (years)		41.22 ± 11.28	39.57 ± 11.52	1.494^t^	0.136
Gender	Male	83 (66.40)	617 (70.76)	0.992	0.319
	Female	42 (33.60)	255 (29.24)		
Blood type	O-Rh+	59 (47.20)	405 (46.44)	0.104	0.991
	A-Rh+	27 (21.60)	196 (22.48)		
	B-Rh+	31 (24.80)	211 (24.20)		
	AB-Rh+	8 (6.40)	60 (6.88)		
BMI (kg/m^2^)		22.01 ± 3.39	22.04 ± 3.55	0.079	0.937
Preoperative dialysis (months)		28.46 ± 22.10	28.57 ± 26.47	−0.747^Z^	0.455
Operation duration (h)		2.86 ± 0.67	2.96 ± 0.92	1.182^t^	0.238
Past Medical History	Coronary artery disease	21 (16.80)	104 (11.93)	2.368	0.124
	Hypertension	114 (91.20)	808 (92.66)	0.335	0.563
	Diabetes	14 (11.20)	97 (11.12)	0.001	0.980
	Anemia	95 (76.00)	629 (72.13)	0.822	0.365
	Hyperlipemia	21 (16.80)	164 (18.81)	0.291	0.589
	Heart failure	8 (6.40)	14 (1.61)	11.646	0.001
	Long-term use of CNI	0 (0)	7 (0.80)	1.011	0.315
	Combined with malignant tumor	2 (1.60)	11 (1.26)	0.097	0.755
Immunosuppressive therapy regimen	Tacrolimus + MPA + glucocorticoid	122 (97.6)	858（98.39）	1.055	0.788
	Cyclosporine + MPA + glucocorticoid	0（0）	1（0.11）		
	CNI + MZR + glucocorticoid	0 (0)	0 (0)		
	CNI + mTOR inhibitor + glucocorticoid	0 (0)	1 (0.11)		
	Other	3 (2.40)	12 (1.38)		
Prevention and treatment of early acute organ rejection after kidney transplantation	None	10 (8.00)	111 (12.73)	2.293	0.130
	Basiliximab/Anti-human T-cell porcine immunoglobulin/Rabbit anti-human thymocyte immunoglobulin	115 (92.00)	761 (87.27)		

By comparing the blood routine levels of the two groups of patients, it was found that the EOS%, HCT, and MCV in the IFIs group were significantly lower than those in the control group (*P* < 0.05). Comparing the liver and kidney function indicators of the two groups of patients, the A/G in the IFIs group were significantly lower than those in the control group, while the GLOB, GGT, BUN, and blood creatinine (Cr) in the IFIs group were significantly higher than those in the control group (*P* < 0.05). Comparing the electrolyte indicators of the two groups of patients, the K + content in the IFIs group was significantly higher than that in the control group, while the Mg + in the IFIs group were significantly lower than those in the control group (*P* < 0.05). Comparing the T lymphocyte subsets of the two groups of patients, the CD4+, and CD4+/CD8 + ratios in the IFIs group were significantly lower than those in the control group (*P* < 0.05, Table 2). In this study, the vast majority of patients (approximately 98%) received tacrolimus as the primary calcineurin inhibitor, while only a small proportion were treated with cyclosporine. Due to this highly unbalanced distribution, stratified analysis between different CNIs was not performed.

**Table 2:** Comparison of laboratory examination indicators between the two groups.

Characteristics	IFIs group (*n* = 125)	Control group (*n* = 872)	t/Z	*P*
WBC (10^9/L)	8.85 ± 3.46	8.83 ± 3.07	0.087^t^	0.931
RBC (10^12/L)	3.13 ± 0.69	3.19 ± 0.69	−0.983^t^	0.326
HGB (g/L)	86.84 ± 18.84	90.20 ± 18.65	−1.885^t^	0.060
PLT (10^9/L)	202.05 ± 82.95	196.59 ± 74.93	−0.947^Z^	0.343
NEU%	75.63 ± 10.81	75.19 ± 15.51	0.308^t^	0.758
LYM%	16.09 ± 10.91	16.62 ± 10.23	−0.993^Z^	0.321
NLR	8.27 ± 8.87	8.31 ± 15.39	−0.920^Z^	0.358
PLR	20.40 ± 25.43	18.32 ± 27.10	−0.948^Z^	0.343
MLR	0.84 ± 1.73	0.66 ± 0.86	−0.655^Z^	0.513
SII	1,678.04 ± 2,364.69	1,489.44 ± 2,562	−1.044^Z^	0.297
MONO%	8.00 ± 8.00	7.36 ± 4.55	0.305^Z^	0.760
EOS%	1.31 ± 1.51	1.92 ± 2.29	−3.544^Z^	<0.001
BASO%	0.23 ± 0.28	0.25 ± 0.52	−1.041^Z^	0.298
HCT (%)	0.27 ± 0.06	0.28 ± 0.06	−2.017^t^	0.044
MCV (fL)	86.45 ± 9.28	88.23 ± 8.34	−2.192^t^	0.029
MCH (pg)	28.03 ± 3.56	28.75 ± 4.22	−1.821^t^	0.069
MCHC (g/L)	323.56 ± 11.68	323.8 ± 12.18	−0.207^t^	0.836
TBIL (*μ*mol/L)	8.95 ± 13.15	7.99 ± 9.29	−0.219^Z^	0.827
DBIL (μmol/L)	4.52 ± 6.71	3.89 ± 7.32	−0.354^Z^	0.724
IBIL (μmol/L)	4.42 ± 6.68	4.10 ± 2.84	−0.878^Z^	0.380
TP (g/L)	60.18 ± 6.43	59.89 ± 6.49	0.473^t^	0.636
ALB (g/L)	37.58 ± 3.91	38.27 ± 4.00	−1.8271^t^	0.068
GLOB (g/L)	22.61 ± 5.17	21.62 ± 4.80	2.136^t^	0.033
A/G	1.75 ± 0.45	1.85 ± 0.41	−2.540^t^	0.011
GGT (U/L)	31.19 ± 25.76	28.85 ± 29.42	−2.412^Z^	0.016
AST (U/L)	17.80 ± 21.06	16.45 ± 15.42	−1.402^Z^	0.161
ALT (U/L)	19.59 ± 19.42	20.83 ± 24.38	−0.085^Z^	0.932
ALP (U/L)	76.83 ± 44.89	78.01 ± 67.82	−0.546^Z^	0.585
BUN (mmol/L)	17.84 ± 10.54	15.19 ± 7.65	−1.995^Z^	0.046
Cr (μmol/L)	214.60 ± 131.45	170.71 ± 106.44	−4.548^Z^	<0.001
UA (μmol/L)	326.53 ± 120.51	322.99 ± 113.96	−0.245^Z^	0.806
K (μmol/L)	4.18 ± 0.59	4.06 ± 0.57	2.139^t^	0.033
Na (μmol/L)	140.43 ± 3.40	140.86 ± 3.31	−1.337^t^	0.182
Cl (μmol/L)	106.96 ± 4.84	108.31 ± 9.47	−1.571^t^	0.117
Ca (g/L)	2.22 ± 0.22	2.23 ± 0.21	−0.561^t^	0.575
Mg (g/L)	0.75 ± 0.14	0.78 ± 0.15	−2.037^t^	0.042
CD3 ^+^ T(%)	65.21 ± 19.87	68.54 ± 17.99	−1.908^t^	0.057
CD4 ^+^ T(%)	37.51 ± 14.48	40.28 ± 13.01	−2.194^t^	0.028
CD8 ^+^ T(%)	25.57 ± 9.86	25.59 ± 8.87	−0.022^t^	0.982
CD4 ^+^ T/CD8 ^+^ T	1.57 ± 0.77	1.79 ± 1.08	−2.218^t^	0.027
FPG (mmol/L)	5.49 ± 2.43	5.31 ± 1.56	1.038^Z^	0.299
TC (mmol/L)	4.11 ± 0.98	4.16 ± 0.95	−0.555^t^	0.579
TG (mmol/L)	1.80 ± 0.85	1.78 ± 0.87	0.370^Z^	0.711
HDL-C (mmol/L)	1.21 ± 0.36	1.22 ± 0.33	−0.316^Z^	0.752
LDL-C (mmol/L)	2.39 ± 0.84	2.43 ± 0.82	−0.158^Z^	0.874

### 3.3 Multivariate analysis of factors influencing the occurrence of IFIs after kidney transplantation

The 13 variables with significant differences identified in the above single-factor analysis (heart failure, EOS%, HCT, MCV, A/G, GLOB, GGT, BUN, Cr, K+, Mg+, CD4 + T, CD4 + T/CD8 + T) were included in the LASSO regression analysis for feature selection. LASSO regression revealed that a total of 12 indicators were non-zero variables (heart failure, EOS%, HCT, MCV, GLOB, GGT, BUN, Cr, K+, Mg+, CD4 + T, CD4 + T/CD8 + T), as shown in Figure 2. After diagnosing for collinearity, the variance inflation factors of these 12 indicators were all < 5, as shown in Table 3. Further, a binary logistic regression (stepwise forward) was employed to analyze the independent factors influencing the occurrence of IFIs after kidney transplantation. The results showed that heart failure (OR = 4.235), low EOS% level (OR = 0.825), high Cr level (OR = 1.003), and low Mg + level (OR = 0.198) were independent risk factors for the occurrence of IFIs after kidney transplantation (*P* < 0.05), as shown in Table 4.

**Figure 2:**
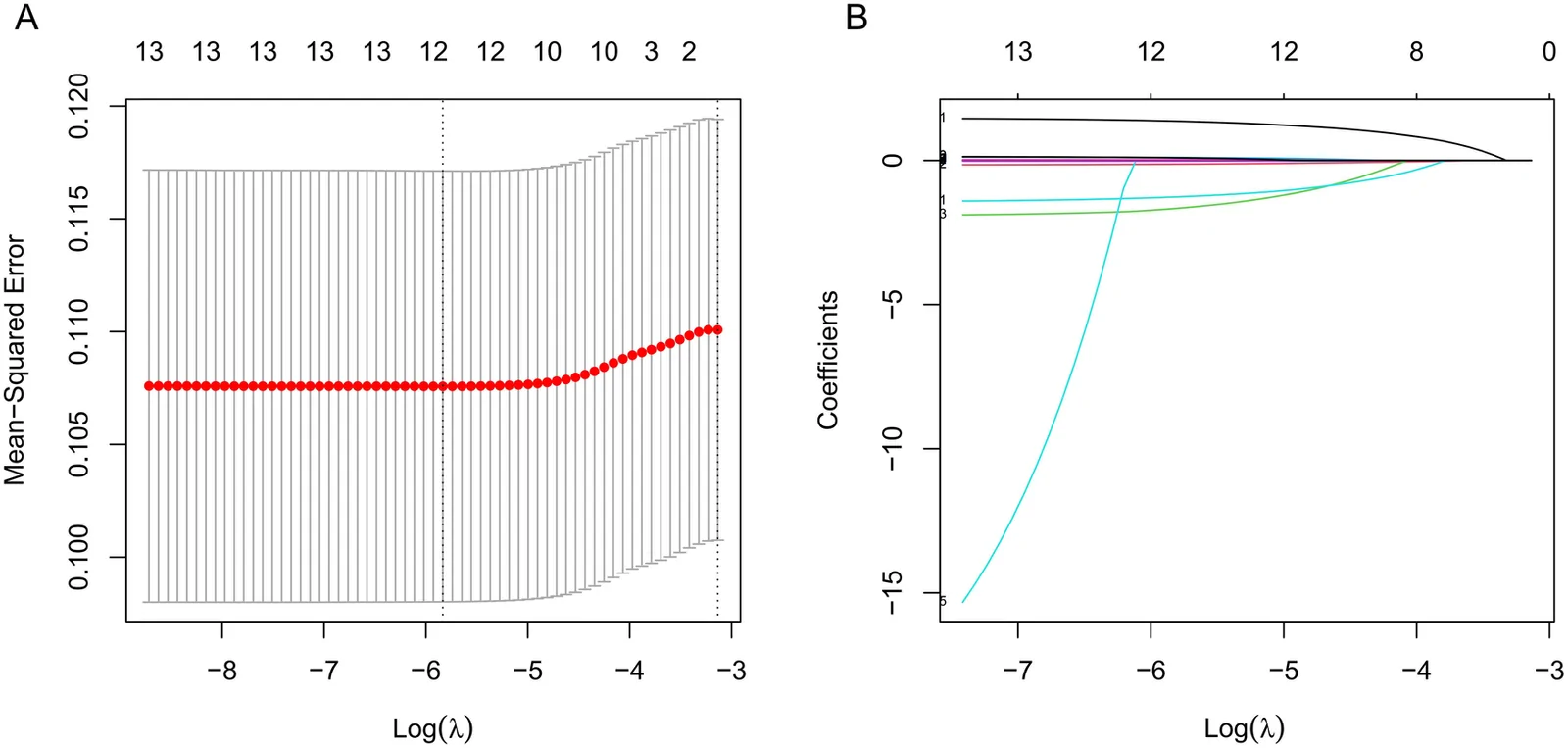
LASSO regression analysis. **(A)** The process of selecting the optimal regularization parameter *λ* for 10-fold cross-validation. When log(*λ*) = −5.8, the model has the minimum partial likelihood bias (*λ*.min), and at this point, there are 12 non-zero coefficient variables. **(B)** The path diagram showing the changes in the coefficients of each variable with log(*λ*). It shows that as *λ* increases, the coefficients of A/G shrink to zero earlier and are excluded, and finally, 12 predictor factors are retained for the subsequent multivariate analysis.

**Table 3:** Multicollinearity diagnosis.

Variables	tolerance	VIF
heart failure	0.990	1.010
EOS%	0.955	1.047
HCT	0.912	1.096
MCV	0.979	1.022
A/G	0.942	1.062
GLOB	0.982	1.018
GGT	0.554	1.805
BUN	0.580	1.725
Cr	0.938	1.066
K	0.921	1.086
Mg	0.606	1.650
CD4 ^+^ T	0.632	1.582
CD4 ^+^ T/CD8 ^+^ T	0.990	1.010

VIF, Variance Inflation Factor, VIF < 5: Collinearity is generally considered acceptable.

**Table 4:** Multivariate logistic regression analysis of IFIs after kidney transplantation.

Step	Indexs	β	S.E.	Wald	P	OR	95%CI low	95%CI upper
step 1	Cr	0.003	0.001	15.245	<0.001	1.003	1.001	1.004
	constant	−2.450	0.169	211.008	<0.001	0.086		
step 2	Heart failure	1.473	0.457	10.401	0.001	4.364	1.782	10.684
	Cr	0.003	0.001	15.700	<0.001	1.003	1.001	1.004
	constant	−2.512	0.172	213.714	<0.001	0.081		
step 3	Heart failure	1.488	0.462	10.372	0.001	4.427	1.790	10.946
	EOS%	−0.192	0.075	6.465	0.011	0.826	0.712	0.957
	Cr	0.002	0.001	11.032	0.001	1.002	1.001	1.004
	constant	−2.142	0.216	98.228	<0.001	0.117		
step 4	Heart failure	1.443	0.464	9.663	0.002	4.235	1.705	10.522
	EOS%	−0.192	0.076	6.377	0.012	0.825	0.711	0.958
	Cr	0.003	0.001	12.959	<0.001	1.003	1.001	1.004
	Mg+	−1.620	0.697	5.408	0.020	0.198	0.051	0.775
	constant	−0.943	0.551	2.930	0.087	0.390		

### 3.4 Prediction model for the occurrence of IFIs after kidney transplantation

After randomly dividing 997 samples into a training set and a validation set in a 7:3 ratio, a prediction model was constructed based on the training set samples (697 in total, 86 with IFIs and 611 controls). Using the above 4 independent risk factors as predictors and IFIs as the observed outcome, a nomogram prediction model was constructed. The model is shown in Figure 3. The model formula is log[P/(1 - P) = −0.631 + 1.471 × Heart Failure - 0.154 × EOS + 0.002 × Cr - 2.039 × Mg. Hosmer and Lemeshow goodness of fit test: X-squared = 8.756, *P*-value = 0.363, it indicates that the model fitting effect is good.

**Figure 3:**
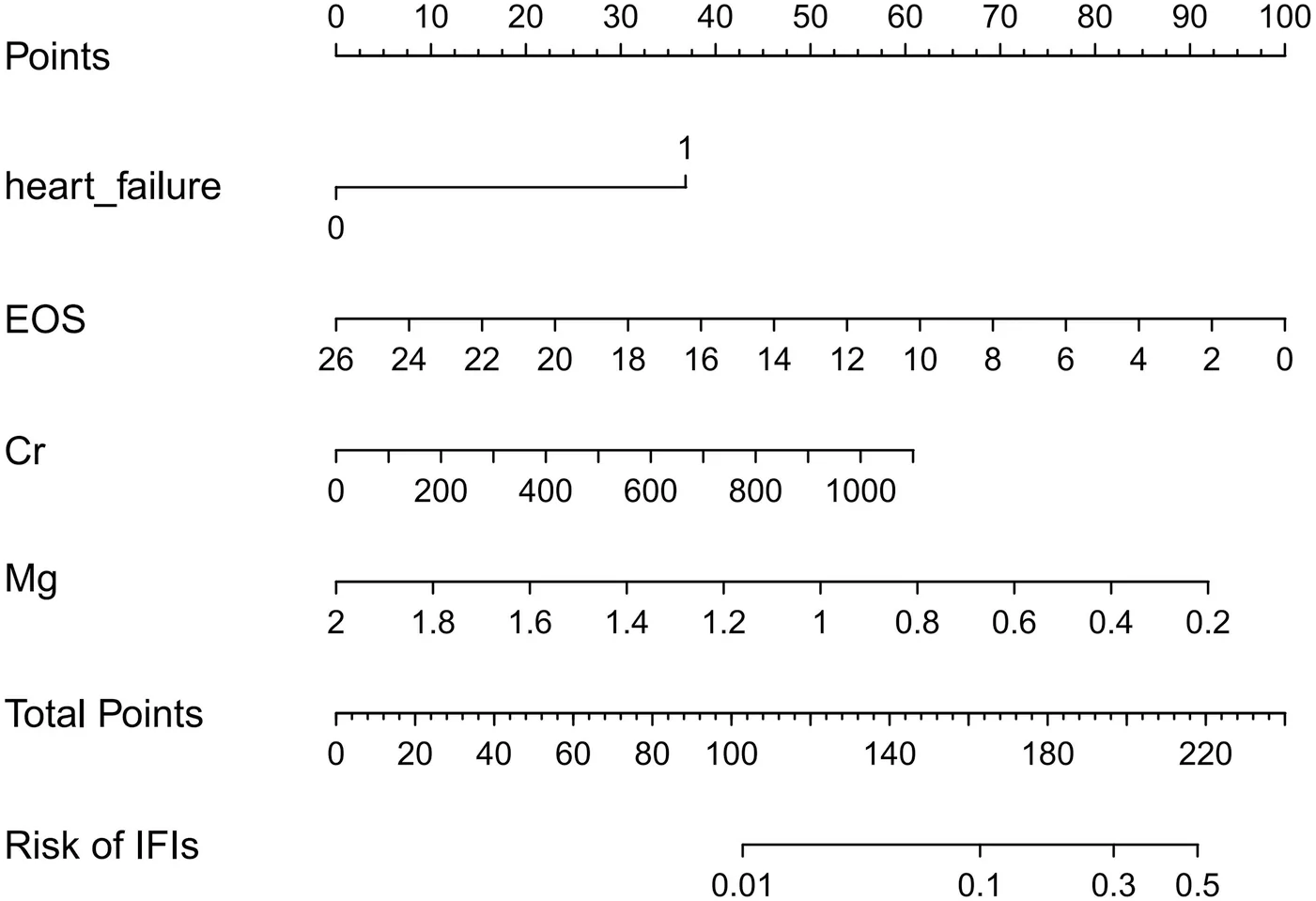
A nomogram model of IFIs occurrence after renal transplantation.

### 3.5 Evaluation of the predictive efficacy of the model

The results of the area under the ROC curve indicated that the AUC of the training set was 0.654 (95% CI: 0.591–0.717), with a specificity of 59.3% and a sensitivity of 65.3% (as shown in Figure 4A). The AUC of the validation set was 0.677(95% CI: 0.583–0.770), with a specificity of 59.0% and a sensitivity of 76.6% (as shown in Figure 4B).

**Figure 4:**
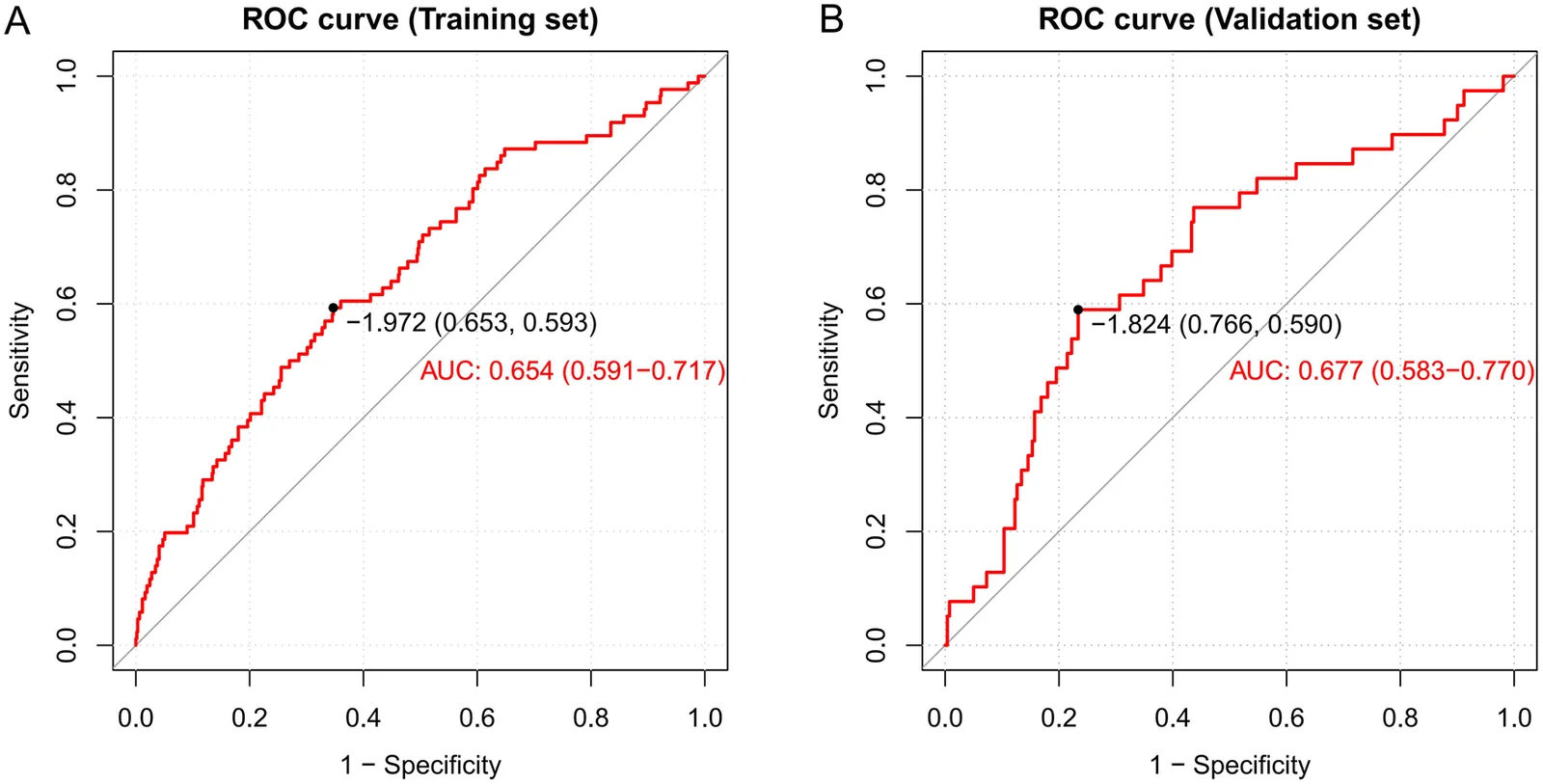
ROC curve for predicting postoperative IFIs in renal transplant recipients. A: Training set; B: Validation set.

The calibration curve results showed that the calibration lines of the training set and the validation set were close to the ideal line, the Brier scores of the training set and the validation set are 0.103 and 0.108 respectively, the calibration slope and calibration intercept of the validation set are 1.1609 and 0.3914, indicating that the model demonstrated good calibration performance with relatively low prediction error. Although the calibration intercept suggested a slight underestimation of the observed risk and the calibration slope indicated a minor tendency toward underfitting, both parameters remained close to the ideal values (intercept = 0 and slope = 1), supporting the robustness and reliability of the model in the validation cohort (as shown in Figures 5A,B).

**Figure 5:**
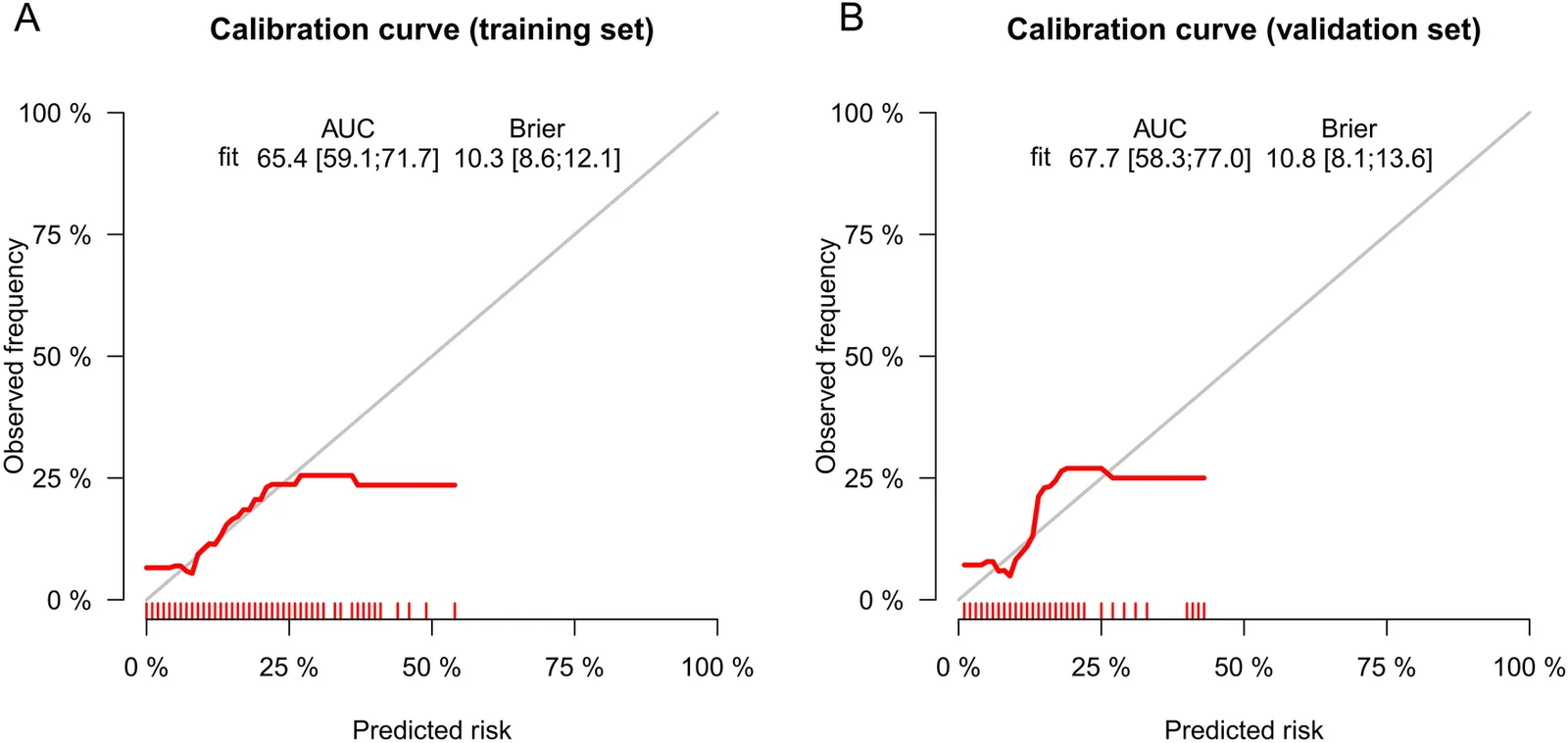
Calibration curve for predicting postoperative IFIs in renal transplant recipients. **(A)** Training set; **(B)** Validation set.

The decision curve analysis results indicated that the threshold of the training set was within the range of 0.2–0.7, and the nomogram model predicted a net benefit rate of > 0 for the occurrence of IFIs in kidney transplant recipients, with the maximum net benefit rate being 0.4 (as shown in Figure 6A). The threshold of the validation set was within the range of 0.15–0.5, and the nomogram model predicted a net benefit rate of > 0 for the occurrence of IFIs in kidney transplant recipients, with the maximum net benefit rate being 0.6, suggesting that the clinical applicability of the model is generally moderate (as shown in Figure 6B).

**Figure 6:**
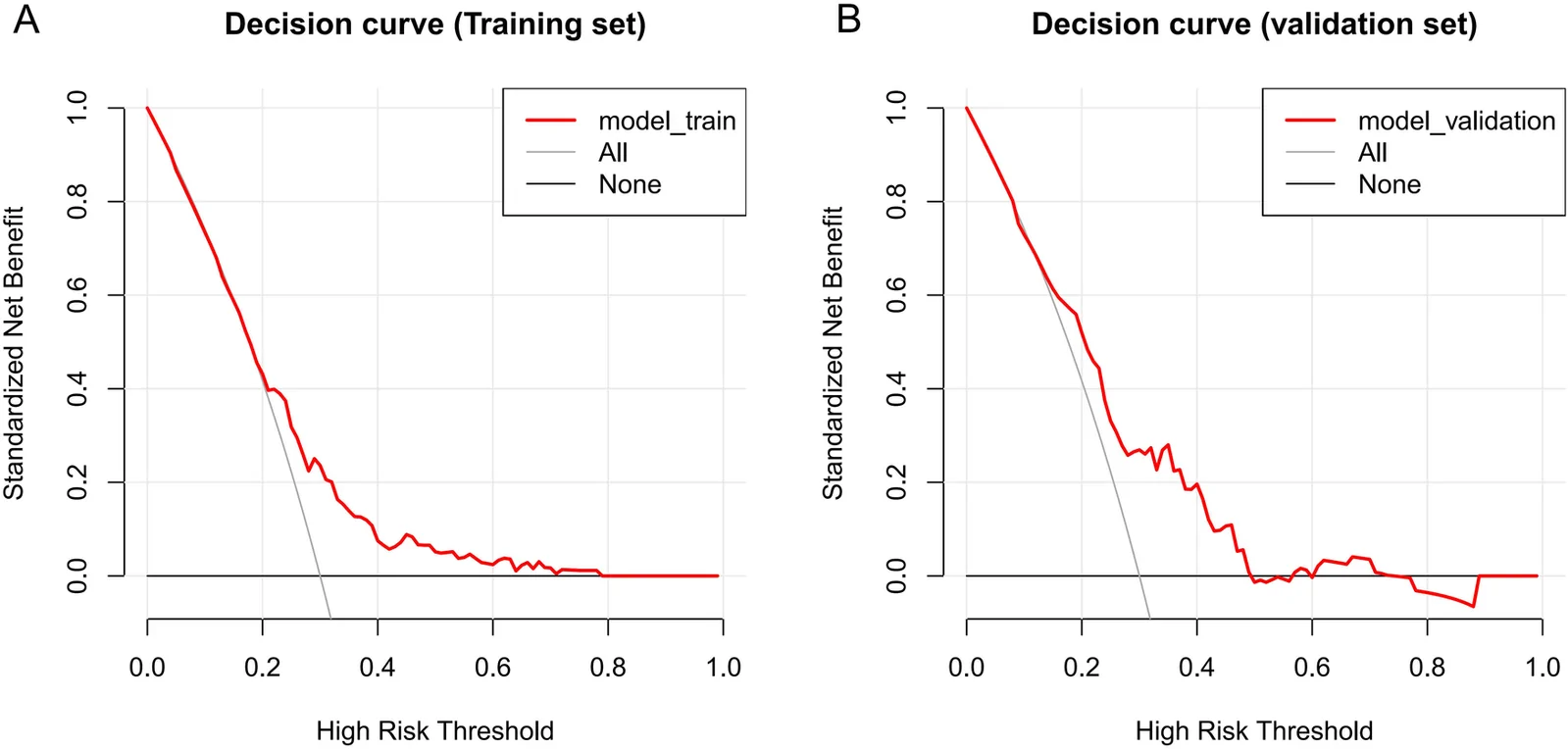
Decision curve for predicting postoperative IFIs in renal transplant recipients. **(A)** Training set **(B)** Validation set.

### 3.6 External validation assessment of the model

In the external validation cohort (Table 5), the nomogram showed satisfactory discrimination and calibration performance. The AUC was 0.700 (95% CI: 602–798), with a calibration slope of 0.8127, calibration intercept of −0.4324, and Brier score of 0.102, indicating moderate discrimination ability and good overall calibration with relatively low prediction error. Although the calibration slope slightly deviated from the ideal value of 1 and the negative calibration intercept suggested a minor overestimation of the predicted risk, the model maintained acceptable calibration performance and demonstrated robustness when applied to an independent external cohort (as shown in Figure 7).

**Table 5:** External validation cohort.

Characteristics	IFIs group (*n* = 27)	Control group (*n* = 196)	χ^2^/Z	*P*
Heart failure	3 (11.11)	0 (0)	22.075	<0.001
EOS%	1.71 ± 1.09	2.18 ± 1.86	−0.686	0.493
Cr	324.81 ± 282.90	263.43 ± 275.34	−2.078	0.038
Mg	0.76 ± 0.11	0.88 ± 0.30	−2.934	0.003

**Figure 7:**
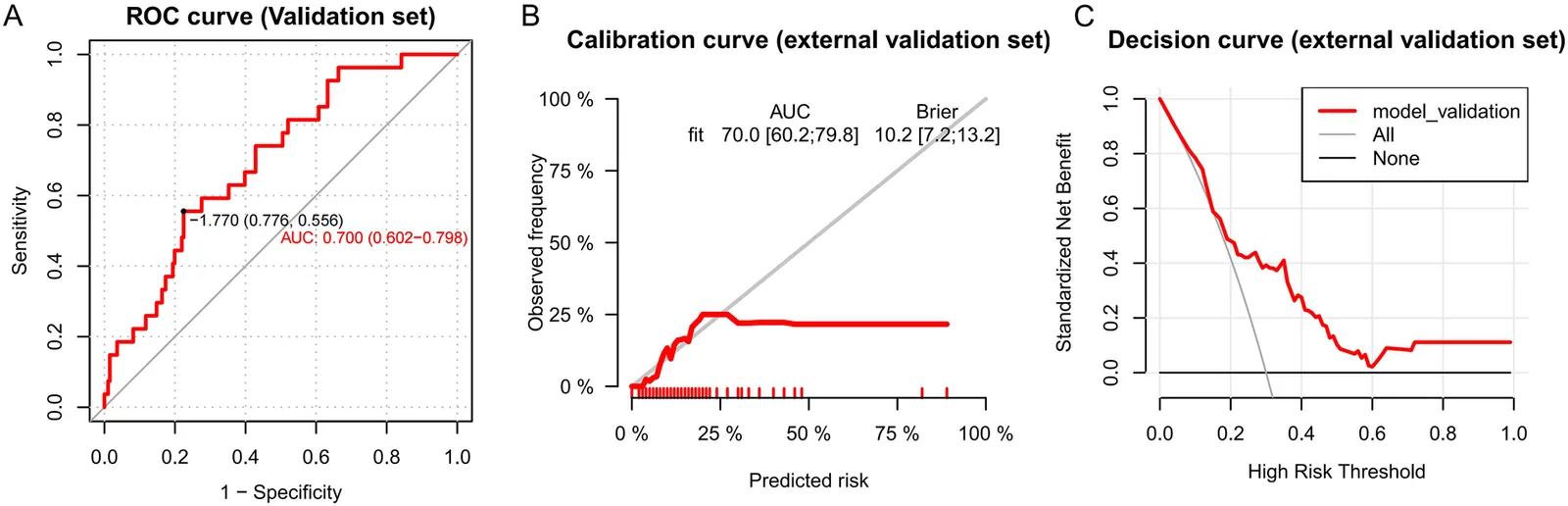
Evaluation of the model's predictive performance on the external validation set. **(A)** ROC curve; **(B)** Calibration curve; **(C)** Decision curve.

## 4 Discussion

For kidney transplant recipients, IFIs are not only a major challenge but also a key factor affecting their survival and prognosis. Understanding the epidemiological characteristics, clinical and laboratory examination features, and risk factors of IFIs in the kidney transplant recipients at this center is of great significance for the prevention and management of IFIs. This study included a total of 125 patients with IFIs after kidney transplantation, and the proportion of males was relatively high. In terms of infection sites, pulmonary infection accounted for the highest proportion (85.93%), which might be related to the long-term use of immunosuppressants by patients after kidney transplantation and the fact that the lungs are open organs that are more susceptible to pathogen invasion. Among the detected strains, the proportion of *Pneumocystis jirovecii* at this center was the highest at 60.8% (76/125), followed by *Aspergillus*, *Candida*, and a few rare infection species such as *Cryptococcus*, *Talaromyces marneffei*, and *Purpureocillium lilacinum*. In the present study, *Pneumocystis jirovecii* accounted for 60.8% of all identified fungal pathogens, representing the predominant etiological agent. This finding differs from previous multicenter studies and conventional clinical observations, in which Candida and Aspergillus species are typically the most frequently reported pathogens. Several factors may explain this discrepancy. First, regional and local epidemiological characteristics may play an important role, as the distribution of fungal pathogens can vary substantially across geographic areas. Second, the specific patient population in our center, including the degree and type of immunosuppression, may predispose to a higher incidence of Pneumocystis infection. Third, differences in prophylactic strategies, particularly the use of trimethoprim–sulfamethoxazole, may significantly influence pathogen distribution. Our center routinely recommends a 3- to 6-month course of sulfamethoxazole at an oral dose of 0.48 g once daily for post-transplant patients. However, approximately 30%–40% of patients with suboptimal renal function recovery after transplantation discontinue sulfonamide prophylaxis upon hospital discharge, which may account for the elevated incidence of PJP in this population. Variations in the duration, dosing, and patient adherence to prophylaxis could lead to differing rates of Pneumocystis infection compared with other studies. The long-term use of tacrolimus—which is associated with a higher risk of Pneumocystis jirovecii pneumonia (PJP) than cyclosporine—in a large proportion of patients at our center (11), together with the application of next-generation sequencing (NGS) for pathogen detection in bronchoalveolar lavage fluid (BALF), has significantly increased the diagnostic positivity rate for PJP (12). Additionally, as a single-center study, our findings may reflect local clinical practice patterns and may not be fully generalizable to broader populations. In terms of infection time, the proportion of IFIs infection occurring within one year after kidney transplantation was 82.40%, indicating that IFIs may occur in the middle and late stages of transplantation, suggesting the need for continuous monitoring of patients' immune status and infection signs during long-term postoperative follow-up (13). Based on the epidemiological characteristics of IFIs at this center, it suggests that clinicians need to pay special attention to the infection risks of *Pneumocystis jirovecii*, *Aspergillus*, and *Candida* in postoperative management, especially the monitoring of infection indicators in the lungs, and optimize the immunosuppressive regimen to reduce the incidence and severity of IFIs.

This study systematically analyzed various potential risk factors by comparing the clinical characteristics and laboratory examination results of the IFIs group and the control group after kidney transplantation. The results showed that heart failure, low EOS%, high Cr level, and low Mg level were important independent risk factors affecting the occurrence of IFIs after kidney transplantation. This study is the first to include low magnesium levels in the analysis, and confirmed that they are independent risk factors for post-kidney transplant infection. This provides a new perspective for clinical practice.

Through the analysis of risk factors, it was found that heart failure is one of the risk factors for IFIs after kidney transplantation. The risk of its occurrence is 4.235 times that of patients without heart failure. Heart failure patients often have multiple underlying diseases, such as diabetes and hypertension, which themselves can have a negative impact on the body's immune function (14). In addition, the systemic circulatory disorder caused by heart failure may affect the distribution and metabolism of drugs in the body, reducing the therapeutic effect of the drugs. Moreover, secondary to heart failure, reduced venous return to the heart leads to increased venous pressure in the liver and portal system, resulting in intestinal congestion that may impair the integrity of the intestinal mucosal barrier. This compromised barrier function increases the risk of microbial and fungal translocation from the gut lumen into the bloodstream (15, 16), thereby contributing to the elevated IFI risk observed in heart failure patients. After kidney transplantation, these factors work together to increase the risk of IFIs and the difficulty of treatment. Therefore, it is necessary to actively control other underlying diseases coexisting in heart failure patients, such as diabetes and hypertension. These diseases may further weaken the immune function of the patients.

In the present study, a lower eosinophil percentage (EOS%) was identified as an independent predictor of IFIs after kidney transplantation. Although eosinophils are traditionally recognized for their roles in allergic inflammation and parasitic infections, accumulating evidence indicates that they also participate in innate immune regulation and host defense against fungal pathogens (17, 18). Eosinophils can contribute to antifungal immunity through the release of cytotoxic granule proteins, production of reactive oxygen species, and modulation of immune cell responses. Therefore, reduced circulating eosinophil levels may reflect impaired antifungal immune surveillance in immunocompromised recipients. During severe systemic inflammation, activation of the hypothalamic–pituitary–adrenal axis and increased endogenous glucocorticoid release can induce eosinophil redistribution and apoptosis, resulting in decreased circulating eosinophil levels (19). Therefore, a reduced EOS% may represent a surrogate marker of a high inflammatory burden and vulnerability to opportunistic infections, including IFIs.

Cr is an important indicator of renal function. High Cr levels usually indicate impaired renal function (20). Moreover, high Cr levels may suggest that the patient has systemic inflammatory response or other complications, which will further increase the risk of invasive fungal infection (21). Studies have shown that the serum creatinine level one month after kidney transplantation is related to long-term prognosis. Patients with serum creatinine levels ≥ 1.52 mg/dL have a significantly increased risk of death (HR = 1.799, *P* = 0.013) (22). This study also shows that high Cr levels are an important risk factor for invasive fungal infection after kidney transplantation. Therefore, in clinical practice, attention should be paid to monitoring the Cr level, and corresponding preventive and treatment measures should be taken according to the specific situation of the patients.

Recent studies have shown that hypomagnesemia is relatively common among patients after kidney transplantation and is significantly associated with the risk of postoperative infection (23). Magnesium, as an important cation, plays a crucial role in cellular metabolism and immune function. At the cellular level, magnesium is an essential cofactor for T-cell activation, proliferation, and cytokine production, including IL-2 and IFN-*γ* synthesis, and is required for immunoglobulin synthesis by B lymphocytes (24). Magnesium deficiency impairs both cellular and humoral immunity by reducing T-cell receptor signaling strength and antibody responses (25). Furthermore, emerging evidence suggests that hypomagnesemia may be associated with an increased risk of acute rejection after kidney transplantation, possibly through its effects on immune cell function and inflammatory responses (26). Beyond its direct immunomodulatory effects, low magnesium levels have been linked to insulin resistance and the development of diabetes mellitus, as well as to cardiovascular diseases including hypertension and arrhythmias—conditions that are prevalent in the kidney transplant population and may further compound infection risk (27, 28). It is also important to note that calcineurin inhibitors and diuretics, which are commonly used in transplant recipients, are well-documented causes of hypomagnesemia through renal magnesium wasting (29). This drug-induced hypomagnesemia not only increases the risk of infection but has also been associated with higher sepsis-related mortality in critically ill patients (30). Therefore, routine monitoring and correction of serum magnesium levels in kidney transplant recipients may represent a simple yet effective strategy to reduce the incidence and severity of IFIs.

Recent studies have shown that hypomagnesemia is relatively common among patients after kidney transplantation and is significantly associated with the risk of postoperative infection (31). Magnesium, as an important cation, plays a crucial role in cellular metabolism and immune function. It participates in the development of immune cells, signal transduction, and the synthesis of immunoglobulins, and is essential for maintaining the immune homeostasis of the body. Van Laecke et al. (32) found that patients with lower serum magnesium levels at the time of transplantation had a significantly increased risk of hospitalization due to severe infection in the first year after surgery (23). The significant correlation between hypomagnesemia and invasive fungal infection after kidney transplantation suggests that monitoring and management of postoperative magnesium levels may be of great significance in reducing the risk of infection.

The predictive model established based on these four independent risk factors demonstrates certain predictive value and can provide an effective tool for the clinical field to identify high-risk patients with IFIs at an early stage. The application of this model helps to optimize prevention strategies and reduce the incidence and severity of IFIs. For instance, by regularly monitoring cardiac function, renal function, electrolyte levels, and metabolic status, combined with individualized risk assessment of patients, targeted intervention measures can be taken in advance, such as adjusting the dosage of immunosuppressants, supplementing magnesium, optimizing heart failure treatment plans, etc. Moreover, this predictive model can also provide new directions for clinical research. As the current research has not yet obtained the permission from other centers, multi-center validation cannot be conducted; the lack of multi-center validation may limit the universality of this model. Future studies can further verify the universality and accuracy of this model in different patient populations and explore more potential risk factors to continuously improve the predictive system. By combining multi-center, prospective research designs, the actual effectiveness of intervention measures based on this model in preventing IFIs can be further evaluated, providing more solid scientific evidence for infection management after kidney transplantation.

## 5 Conclusion

This study retrospectively analyzed the high-risk factors of IFIs after kidney transplantation. The results showed that heart failure, low EOS%, high Cr level, and low Mg level were important independent risk factors affecting the occurrence of IFIs after kidney transplantation. The predictive model based on these risk factors has great guiding value for clinical practice. In the future, it needs to be verified by a larger sample in multiple centers, and it is expected to accurately identify high-risk groups and provide a basis for clinical prevention.

## Data Availability

The raw data supporting the conclusions of this article will be made available by the authors, without undue reservation.
